# MECP2 Dysfunction in Rett Syndrome: Molecular Mechanisms, Multisystem Pathology, and Emerging Therapeutic Strategies

**DOI:** 10.3390/ijms26178277

**Published:** 2025-08-26

**Authors:** Gyutae Choi, Sanghyo Lee, Seungjae Yoo, Jeung Tae Do

**Affiliations:** 1 Department of Stem Cell and Regenerative Biotechnology, KU Institute of Technology, Konkuk University, Seoul 05029, Republic of Korea; ch08david@naver.com (G.C.); lsh00119@naver.com (S.L.); tmdwo323@naver.com (S.Y.); 2 3D Tissue Culture Research Center, Konkuk University, Seoul 05029, Republic of Korea

**Keywords:** Rett syndrome, MECP2, transcriptional regulation, epigenetics, AAV gene therapy, X chromosome inactivation, neuronal dysfunction, glial pathology

## Abstract

Rett syndrome is a severe neurodevelopmental disorder that occurs primarily in females and is caused by mutations in the methyl-CpG-binding protein 2 (*MECP2*) gene located on the X chromosome. Though MECP2 acts as a representative transcriptional regulator and affects gene expression both directly and indirectly, a complete understanding of this disease and the treatment mechanism has not been established yet. MECP2 plays a particularly important role in synaptic development, neuronal maturation, and epigenetic regulation in the brain. In this study, we summarize the molecular structure of MECP2, mutation-specific pathogenesis, and the role of MECP2 in regulating chromatin remodeling, RNA splicing, and miRNA processing to provide a comprehensive understanding of Rett syndrome. Additionally, we describe abnormal phenotypes manifested in various brain regions and other tissues owing to MECP2 dysfunction. Finally, we discuss current and future therapeutic approaches, including AAV-based gene therapy, RNA editing, X chromosome reactivation, and pharmacological interventions. Understanding the diverse functions and pathological mechanisms of MECP2 provides an important foundation for developing targeted therapies for Rett syndrome.

## 1. Introduction

Rett syndrome is a genetic disease that mainly occurs in females and is caused by a mutation in the methyl-CpG-binding protein 2 (*MECP2*) gene located on the X chromosome (Xq28) in 95% cases [1]. In other cases, a Rett syndrome-like phenotype arises owing to mutations in the *CDKL5* and *FOXG1* genes [2,3]. Rett syndrome is regarded as one of the most common genetic causes of severe cognitive disability following Down syndrome, and the incidence rate is high (1 in 10,000–15,000) [4]. Representative symptoms include stereotypical hand movements without purpose, seizures, autonomic nervous system instability, and hyperventilation [5,6].

To date, 925 *MECP2* variants have been identified, with 535 being pathogenic, and these can be confirmed in RettBASE [7]. Eight common pathogenic variants (R168X, R255X, R270X, R294X, R106W, R133C, T158M, and R306C) have been identified, with a C > T single-nucleotide change occurring in approximately 60–70% females with Rett syndrome [8,9,10].

Most patients with Rett syndrome have a de novo mutation in the paternally inherited X chromosome, which largely accounts for the predominance of the disorder in females [11,12,13]. Although rare, Rett syndrome can also occur in males with *MECP2* mutations; however, these cases are typically associated with severe phenotypes, and the affected individuals often succumb to fatal encephalopathy before the age of two [14,15,16]. Even though *MECP2* mutations can occur on both X chromosomes in females, such biallelic mutations cause embryonic lethality during fetal development in mouse models [17]. Therefore, patients with Rett syndrome typically carry a heterozygous *MECP2* mutation on one of the two X chromosomes. The clinical manifestations of Rett syndrome are primarily ascribed to aberrations in synaptic structure and neuron function, which are regarded as central neuropathological mechanisms underlying the etiology of Rett syndrome.

## 2. X Chromosome Inactivation and MECP2 Mosaicism in Rett Syndrome Pathogenesis

**Figure 1:**
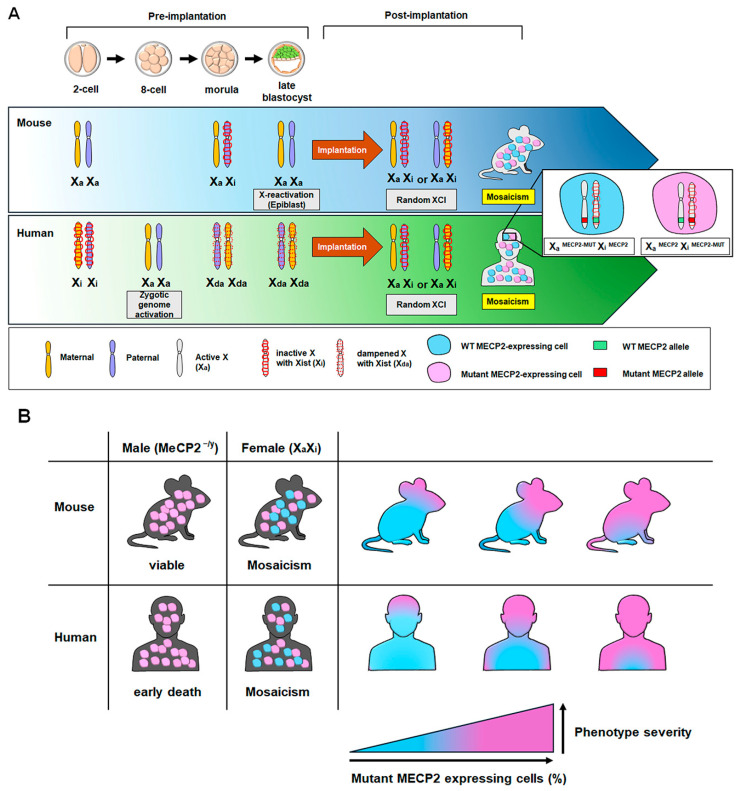
Regulation of X chromosome expression during early embryonic development and the effects of methyl-CpG-binding protein 2 (MECP2) mutations in mice and humans. (**A**) In mice, paternal X chromosome inactivation occurred during preimplantation development during the preimplantation stages as the embryo develops into a blastocyst. X chromosome reactivation occurs only in the epiblast of the late blastocyst stage. In humans, zygotic genome activation takes place at the 8-cell stage and X chromosome dampening begins at the morula stage. In the post-implantation stage, both mouse and human embryos exhibit inactivation of either the maternal or paternal X chromosome, which leads to a mosaic pattern of X chromosome expression. (**B**) The effects of MECP2 mutations differ depending on sex in both mice and humans. Male mice and humans with a MECP2 mutation (*MECP2*^−/y^) show different phenotypes. In females, the severity of the phenotype increases when the proportion of cells expressing the mutant *MECP2* allele becomes higher; Cells expressing mutant MECP2 (pink); cells expressing normal MECP2 (light blue).

Understanding the random X chromosome inactivation (XCI) that occurs during early embryonic development is crucial for understanding Rett syndrome phenotype severity. The choice of which X chromosome to inactivate occurs randomly in vivo in humans and mice [18]. Unlike in mice, where imprinted XCI initiates early during embryogenesis, canonical XCI does not occur in human preimplantation embryos. Instead, both X chromosomes remain transcriptionally active but exhibit reduced expression levels, a phenomenon referred to as X chromosome dampening [19]. Random XCI gradually occurs post-implantation, with one of the two X chromosomes undergoing inactivation [19,20]. Therefore, either the paternal or the maternal X chromosome becomes inactivated in the cells comprising the body, leading to the coexistence of both cell types [18,21]. Thus, patients with Rett syndrome include cells with a *MECP2* mutation (^MECP2-MUT^) on the Xi (Xa^MECP2^ Xi^MECP-MUT^; Xa: active X chromosome, Xi: inactive X chromosome) and cells with a *MECP2* mutation on the Xa (Xa^MECP2-MUT^ Xi^MECP2^) (Figure 1A). The state in which these two types of cells are mixed is called mosaicism, and the more cells expressing mutant MECP2, the more severe the Rett syndrome phenotype appears [22] (Figure 1B).

Due to limited access to post-mortem human brain tissues and patient-derived neurons, mouse models have been widely employed to study MECP2 and Rett syndrome pathogenesis in vivo [23]. However, in mouse Rett syndrome model, females exhibit non-random XCI with preferential expression of WT *Mecp2* allele. *Mecp2*^−/y^ male mice are viable but display neurological impairments [17,24,25]. On the contrary, in human females with Rett syndrome, the severity of the symptom varies depending on mosaicism ratio between cells with Xa^MECP2^ Xi^MECP-MUT^ and Xa^MECP2-MUT^ Xi^MECP2^ through random XCI [26,27,28,29]. Moreover, human males carrying MECP2 mutation typically experience early lethality owing to severe encephalopathy (Figure 1B). Additionally, in mouse Rett syndrome models, symptom onset occurs in adulthood, whereas in humans, symptom onset occurs in childhood [17]. Although the *Mecp2* mutant mouse model mimics the main characteristics of patients with Rett syndrome, it is evaluated as underrepresenting actual patients [30,31]. These differences may make it difficult to analyze the exact pathogenesis of Rett syndrome in mice. Therefore, in vitro studies using human pluripotent stem cells, derived from patients with Rett syndrome or genetically modified MECP2 mutation, offer a suitable platform for modeling Rett syndrome.

## 3. Molecular Mechanism of MECP2 Function

### 3.1. MECP2 Structure and Its Mechanism of Gene Expression Regulation

**Figure 2:**
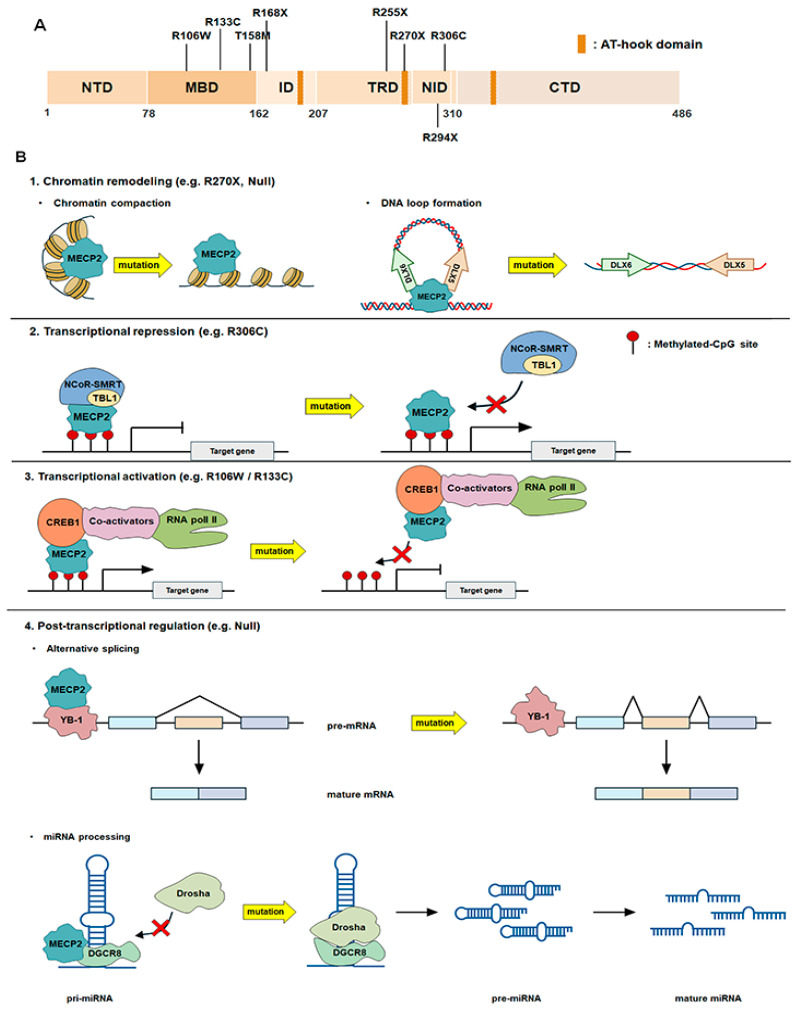
Protein structure of methyl-CpG-binding protein 2 (MECP2) and gene regulation mechanisms. (**A**) Schematic of MECP2 protein and common pathogenic mutations. MECP2 has functional domains including a methyl-CpG-binding domain (MBD), an intervening domain (ID), a transcriptional repression domain (TRD), an NCoR–SMRT interaction domain (NID), and three AT-hook domains. NTD, N-terminal domain; CTD, C-terminal domain. (**B**) Mechanisms of MECP2-mediated gene regulation under normal and mutant conditions. (1) Chromatin compaction and DNA loop formation—Normal MECP2 binds methylated and unmethylated DNA, compacting chromatin in a histone H1-like manner, and forms DNA loops at loci such as DLX5–DLX6 to induce biallelic expression. Mutations in the AT-hook 2 region (e.g., R270X, G273X) impair this chromatin compaction. G273X retains partial function relative to R270X, indicating that amino acids 270–272 are important for AT-rich DNA binding. (2) Co-repressor recruitment—MECP2 interacts with NCoR and SMRT via the NID to repress transcription. The R306C mutation disrupts NCoR–SMRT interaction despite intact DNA binding, leading to partial loss of repression. (3) Transcriptional activation—MECP2 can also activate gene expression by recruiting CREB1 to target promoters. MBD mutations (e.g., R106W, R133C abolish DNA binding, severely altering gene expression. (4) Post-transcriptional regulation—MECP2 interacts with splicing factors such as YB-1 to influence alternative splicing. MECP2 binds DGCR8, inhibiting Drosha–DGCR8 complex formation to repress miRNA processing, while loss of MECP2 elevates miRNA levels in certain brain regions. In null mutations, MECP2 expression is completely absent, resulting in loss of all regulatory mechanisms described above.

MECP2 is a major transcriptional regulator for neuronal maturation that binds to methylated DNA and acts as a global transcriptional regulator, modulating gene activation and repression, thereby influencing cellular phenotypes [5,32]. It also plays a role in regulating mRNA splicing and miRNA biogenesis [33,34,35,36]. MECP2 protein consist of (1) N-terminal domain (NTD), (2) methyl-CpG-binding domain (MBD), (3) intervening domain (ID), (4) transcriptional repression domain (TRD), (5) C-terminal domain (CTD), and (6) three AT-hooks (each located in the ID, TRD, and CTD) (Figure 2A) [37]. The MBD of MECP2 binds to DNA and regulates gene expression through various mechanisms (Figure 2B). First, MECP2 modulates chromatin compaction and DNA loop generation [38]. MECP2 binds to both methylated and non-methylated DNA and regulates genes by inducing chromatin compaction in a manner similar to histone H1 (Figure 2B) [39,40]. Furthermore, MECP2 attaches to the *DLX5*-*DLX6* locus, an imprinted gene cluster, forming a DNA loop to induce biallelic expression (Figure 2B) [41]. Second, MECP2 regulates gene expression by interacting with co-repressors such as histone deacetylases (HDACs), switch-independent 3A (SIN3A), nuclear receptor co-repressor (NCoR), and silencing mediator of retinoic acid and thyroid hormone receptor (SMRT) (Figure 2B) [42,43]. Located in the TRD of MECP2, the NCoR–SMRT interaction domain (NID) mediates MECP2-dependent repression of a broad set of genes [44]. Third, although MECP2 is primarily a transcriptional repressor, it can also activate transcription by activating the target gene promoter via cAMP-responsive element-binding protein 1 (CREB1) (Figure 2B) [45]. In the absence of MECP2, the number of downregulated genes exceeds that of upregulated genes, suggesting that many proteins are positively regulated by MECP2 [46]. Fourth, MECP2 can also bind to RNA, regulating alternative splicing (Figure 2B) [33,36]. The Gene ontology (GO) analysis of MECP2-interacting proteins revealed an enrichment in splicing-related functions, suggesting a role for MECP2 in the post-transcriptional gene expression regulation [33,47]. YB-1, which interacts with MECP2, is a Y-box transcription factor that regulates the transcription of various target genes and binds to mRNA to influence alternative splicing and translation (Figure 2B) [34,48]. Fifth, MECP2 directly interacts with DiGeorge syndrome critical region 8 (DGCR8) and prevents the assembly of the Drosha–DGCR8 complex, a crucial step in miRNA processing, leading to reduced miRNA production (Figure 2B) [36]. In *Mecp2*-null mice, miRNA production is significantly elevated in the hippocampus, suggesting a repressive role for MECP2 in miRNA biogenesis within this brain region [36,49].

### 3.2. MECP2 Mutations Affecting Gene Expression

To understand the pathology of Rett syndrome, the location of mutations in MECP2 affects the brain abnormality types and specific genes and proteins that are regulated should be recognized [50,51]. MECP2-mediated gene regulation is influenced by the specific location of the mutation (such as MBD, TRD, and null) [52] (Figure 2B). (1) In the case of R270X, where a mutation occurs at AT-hook 2 within the TRD and the MBD of MECP2 is intact, MECP2 cannot properly modify chromatin structure despite its preserved DNA-binding ability. The G273X mutant mouse, which has a distance of three amino acids from R270X at the end of AT-hook 2, exhibits a longer lifespan and more efficient chromatin compaction than the R270X mouse [53]. This evidence suggests that amino acids 270–272 in MECP2 are crucial for binding to the AT-rich DNA regions and that the AT-hook domain plays an important role in chromatin modification [53,54]. Furthermore, owing to premature termination, MECP2 lacks the NID, preventing it from binding to the NCoR–SMRT complex. (2) The MECP2 R306C mutation is a representative missense mutation that damages the interaction between MECP2 and NCoR–SMRT co-repressor, thus disrupting the inhibitory role [55]. In this case, the MBD remains intact and allows MECP2 to bind to chromatin; however, the mutation in the NID region impairs its interaction with co-repressor complexes, compromising transcriptional regulation. The resulting phenotype is milder than mutations that entirely abolish DNA binding [56]. (3) When a mutation occurs in the MBD, such as R106W, R133C, and T158M, MECP2 loses its DNA-binding ability and thus cannot regulate gene expression. In the case of R106W and R133C mutations, although the interactions with NID, CTD, and RNA polymerase II are normal, the mutant MECP2 fails to bind to the transcription start site (TSS) of DNA, extensively altering the expression of related genes [57]. Mutations in the MBD and NID, such as T158M and R306C, retain partial MECP2 function, as evidenced by the milder severity in patients with Rett syndrome [58,59]. Therefore, residual interactions with mSin3a, NCoR, and AT-hooks may contribute to this remaining function of MECP2 [53,60]. (4) Since MECP2 is not expressed in the null model, the regulatory mechanism is absent.

### 3.3. Post-Translational Modification Regulation by MECP2

#### 3.3.1. Histone Deacetylation and Chromatin Remodeling via MECP2–HDAC Interactions

MECP2 interacts with DNA to form a complex with HDAC, regulating gene expression, and competes with histone H1 for nucleosome binding, contributing to chromatin structure and gene expression [39]. Alterations in the global chromatin state caused by MECP2 deficiency affect the expression patterns of crucial neuron-specific genes. Additionally, MECP2 deficiency hyperacetylates Histone H3, a crucial histone protein involved in the epigenetic regulation of gene expression through histone modification, in the cerebrum, cerebellum, and spleen of *Mecp2*^308/Y^ mice [30,39]. H3 acetylation at lysine residues K9, K14, K18, K23, K27, and K36 can decondense the nucleosome structure, forming euchromatin and potentially activating previously repressed genes [61,62,63,64,65]. CREB–CBP and SIRT1 are representative examples of chromatin-modifying enzymes that function as histone acetyltransferase (HAT) and HDACs, respectively [66,67]. Taken together, MECP2 regulates gene expression in mature neurons through interactions with histone-modifying enzymes and alters chromatin structure through its histone H1-like role.

#### 3.3.2. Histone Methylation

MECP2 is closely associated with histone methylation, as evidenced by its involvement in histone H3K9 methylation during *IL6* regulation [68]. DNA methylation plays a more dominant role in *IL6* silencing than histone deacetylation. Notably, MECP2 and H3K9 methylation are both enriched at methylated DNA regions, indicating that MECP2 binds to methylated DNA and induces H3K9 methylation to maintain a repressive chromatin state. Consistent with this, the brain cells showed higher DNA methylation levels than other tissues, which may enhance the binding affinity of MECP2 to regions marked by H3K9 and H3K27 methylation [69].

MECP2 was first shown to bind to 5-methylcytosine (5mC) through its MBD. However, subsequent studies have revealed that it also has high affinity for non-CpG methylation (mCH) and 5-hydroxymethylcytosine (5hmC) [70,71,72]. In addition, MECP2 preferentially binds to GC-rich DNA regions, and this binding becomes even stronger when histone H3K27me3 is present [73]. This suggests that histone methylation facilitates MECP2-mediated transcriptional regulation of gene expression.

#### 3.3.3. DNA Methylation

MECP2 directly binds to Dnmt1 to form a complex that binds to hemimethylated DNA, thereby contributing to DNA methylation maintenance during replication [74]. In mouse embryonic stem cells (ESCs), despite the absence of Dnmt1, Dnmt3a, and Dnmt3b, Mecp2 continues to bind chromatin [75]. Since MECP2 can still associate with DNA without these DNA methyltransferases, whether MECP2 directly contributes to DNA methylation maintenance should be further studied.

#### 3.3.4. MECP2 Phosphorylation and SUMOylation

Depending on the specific mutation type in MECP2, the phosphorylation pattern of MECP2 and its interaction with related proteins can vary. Phosphorylation mapping of MECP2 has identified Ser86, Ser274, Thr308, and Ser421 as critical sites [76]. Phosphorylation at these sites is induced by neuronal activity, brain-derived neurotrophic factor (BDNF), and cAMP signaling pathways, functioning as key epigenetic regulation mediators [76]. Specifically, BDNF induces Ser86 and Ser274 phosphorylation in MECP2, while Thr308 and Ser421 phosphorylation in MECP2 is mediated by calcium/calmodulin-dependent protein kinase IV (CaMKIV), which is activated by membrane depolarization [76,77,78]. Thr308 phosphorylation in MECP2 disrupts the interaction between the MECP2 NID and the NCoR complex, thereby inhibiting MECP2’s ability to repress transcription [44]. However, the MECP2 missense mutation R306C prevents Thr308 phosphorylation, making it epigenetically impossible to regulate specific genes [76]. In the T308A mutant mouse brain, the mRNA expression levels of *Npas4* and *Bdnf*, which are essential for inhibitory synapse maturation and plasticity, were significantly decreased. This was accompanied by brain weight loss and motor coordination deficits. Interestingly, treating the T308A mouse with the GABA_A_ receptor antagonist pentylenetetrazol (PTZ) reduced seizures [79,80].

Ser421 phosphorylation in MECP2 is closely associated with dendritic growth, spine maturation, and *BDNF* expression, which are crucial for development of neuronal dendrites and inhibitory synapses in the cortex. This event is also linked to activity-dependent regulation, playing an essential role in establishing connectivity within the nervous system [81,82].

Ser80 of Mecp2 is phosphorylated in resting neurons, but becomes dephosphorylated upon action potentials [83]. Active neurons exhibit Ser80 dephosphorylation but Ser421 phosphorylation. The absence of phosphorylation owing to Mecp2 Ser80 mutation increases the expression levels of Rab3d, Vamp3, and Igsf4b [84]. Rab3d plays a role in the vesicular release machinery [83,85], Vamp3 facilitates SNARE-dependent exocytosis in astrocytes [86], and Igsf4b is a nerve tissue-specific cell adhesion molecule crucial for axon–glia interaction in myelination [87], suggesting that Ser80 phosphorylation in Mecp2 plays a critical role in repressing the expression of genes involved in synaptic vesicle release, exocytosis, and axon–glia interactions, thereby regulating the neuronal activity and myelination. Ser164 phosphorylation in Mecp2 is regulated during neurodevelopment in the brain. This event significantly impairs the DNA-binding affinity of Mecp2 and influences its interaction with nucleosomes and chromatin [83,88]. These data indicate that Mecp2 phosphorylation may profoundly affect brain maturation processes.

Ser421 and Thr308 phosphorylation in MECP2 promotes its SUMOylation, which can be induced by NMDA, IGF-1, and corticotropic releasing factor (CRF) in the rat brain [89]. Mecp2 SUMOylation plays a critical role in enhancing Bdnf mRNA expression by releasing CREB from the repressor complex (MECP2–Sin3a–HDAC1–CREB) [90]. Furthermore, impairments in Mecp2 SUMOylation contribute to deficits in social interaction, long-term memory (also called long-term potentiation, LTP) and synaptic plasticity [89]. Among MECP2 mutations, SUMOylation is significantly reduced in R106W, R133C, P152A, T158M, R306C, and P3mecp76R [91]. The deficit in SUMOylation is attributed to reduced expression of *IGF-2*, *WNT6*, and *WNT5b* genes, aligning with findings in the etiology of patients with Rett syndrome [89,92,93].

## 4. Nervous Systems Affected by MECP2 Mutations

Since Rett syndrome predominantly affects the nervous system, this review explores the brain regions and associated cell types, including neurons and glial cells, that are impacted by Rett syndrome or MECP2 mutations. In addition, we discuss the role of MECP2 in neural development and its contribution to Rett syndrome pathophysiology.

### 4.1. MECP2 Function in Early Neurogenesis

Early studies on MECP2 primarily investigated its role in neuronal maturation, with functional studies focusing on postnatal processes such as synapse formation and dendritic development [81,94,95,96]. In general, MECP2 does not affect neural progenitor cells (NPCs) during development and is expressed more in the nuclei of differentiated neurons than in glial cells [97,98,99,100]. However, MECP2 influences the fate of neural stem cells (NSCs)/NPCs to differentiate into neurons and glial cells [101]. The absence of MECP2 in NSCs/NPCs results in deficits of mature miR-199a. Therefore, *Smad1*—normally repressed by miR-199a—is upregulated, which in turn activates the downstream BMP4–Smad1 signaling pathway, triggering the shift from neuronal to astrocytic differentiation from NSCs/NPCs [101,102]. Belichenko et al. also reported decreased neurons and increased glial cells in patients with Rett syndrome [103]. Therefore, MECP2 expression loss decreases the number of mature neurons during development and, instead, increases the number of glial cells.

However, MECP2 deficiency in NSCs/NPCs can affect the differentiation and proliferation by increasing miR-199a and miR-214 expression, thereby regulating the expression of downstream target proteins [102]. Both miR-199a and miR-214 are important in regulating early neural differentiation and prenatal neurogenesis. miR-199a targets and inhibits *PAK4*, a serine/threonine kinase regulated by Rho family GTPases that activates the mitogen-activated protein kinase signaling pathway. PAK4 inhibition subsequently suppresses the ERK pathway [104]. In the absence of MECP2, miR-214 increases. This miRNA targets and suppresses *PTEN*, a negative regulator of AKT signaling, thereby enhancing AKT activation [105,106]. In general, ERK promotes neural differentiation in embryonic brain development and AKT induces NPC proliferation and survival [107,108]. In vivo studies have also demonstrated increased miR-199b-5p expression in the brains of adult *Mecp2*-mutant mice [49]. Additionally, the BMP/Smad signaling pathway promotes primary miRNA (pri-miRNA) processing [109]. Moreover, Rett syndrome brain organoids showed elevated *BMP4* mRNA expression levels [102]. Taken together, these findings indicate that MECP2 deficiency disrupts neural development by upregulating miRNAs that modulate ERK and AKT signaling pathways. Additionally, enhanced BMP/Smad signaling may promote miRNA processing, further influencing neural differentiation and proliferation.

The expressions of specific miRNAs, such as miR-199a, vary depending on the location and type of *MECP2* mutation or deletion. For example, increased miR-199a was observed in 316C > T missense mutation and 705delG in *MECP2* [102], but decreased miR-199a was observed in 806delG [101]. Further research on the regulation of additional miRNAs in the context of *MECP2* mutation is needed.

### 4.2. Neuronal Defects in Cerebral Cortex

#### 4.2.1. Clinical Alteration of Brain Structure in Rett Syndrome

The resting-state functional MRI (rs-fMRI) analysis demonstrated that the brain size of patients with Rett syndrome was smaller than that of healthy individuals [110]. The greatest reduction was observed in the frontal lobe, and notably, the dorsal attention network (DAN), which is composed of connections between the frontal and posterior parietal cortices, showed decreased functional connectivity [111,112]. Since DAN maturation is essential for developing cognitive abilities and concentration [113], abnormal connections within the DAN may decrease concentration [114]. Accordingly, patients with Rett syndrome exhibit impaired selective focus and concentration, which may be attributed to decreased functional connectivity of the DAN. Similarly, the reduced connectivity of the corpus callosum contributes to the decreased DAN functionality in humans belonging to the autism spectrum [110].

#### 4.2.2. Clinical Alteration of Neurons in Rett Syndrome

Clinical characteristics of neurons in patients with Rett syndrome include smaller cell bodies, reduced dendritic arborization, short and sparse dendrites, and reduced phenotypes of excitatory glutamatergic, inhibitory GABAergic, and monoaminergic neurotransmission [52,115,116,117,118]. MECP2 is crucial for synapse maturation and neurotransmitter activity regulation in neurons [119,120]. Notably, reduced MECP2 expression has been associated with decreased dendritic complexity, as evidenced by reduced spine density and short spine length, which are critical for synaptic structure formation [121,122]. Moreover, the dendritic length of pyramidal neurons in the frontal motor cortex is significantly reduced in patients with Rett syndrome [119]. In particular, the dendrites of pyramidal neurons in layers 3 and 5 of the frontal, motor, and inferior temporal regions, as well as the basal dendrites of layer 4 in the subiculum, are selectively and significantly reduced [123,124]. In a very rare case, a clinical study described a male proband with a *MECP2* mutation who died from central respiratory failure at 15 months of age. The brain was small, with reduced frontal and temporal lobes, a thin corpus callosum, and a marked reduction in synaptic vesicles in the cerebellum and spinal cord. As observed in female cases, the dendritic tree of pyramidal neurons was significantly reduced in cortical layers 3 and 5 of the frontal and temporal lobes [15].

#### 4.2.3. MECP2-Regulated Genes Involved in Neuronal Function

MECP2 is a global transcriptional regulator in neurons. It exerts both repressive and activating effects on target genes that control synaptic transmission, neuronal development, and metabolic regulation. Disruption of MECP2 function in Rett syndrome alters excitatory and inhibitory neurotransmission, impairing neuronal network activity. These alterations are believed to underlie key features of the disorder, including cognitive, behavioral, and motor deficits.

**Excitatory Neurotransmission-Related Genes**

Key excitatory neurotransmission-related genes include *BDNF*, serum/glucocorticoid-regulated kinase 1 (*SGK1*), and *IGFBP3*. *BDNF* regulates neuronal growth and is considered an important neurite outgrowth and synapse formation regulator [125,126]. *Mecp2* knockdown reduces *BDNF* expression at glutamatergic synapses, delaying neuronal growth and maturation, but neuronal growth can be restored upon introduction of *BDNF* [127].

*SGK1* is important for learning, memory, synaptic plasticity in neurons of the hippocampus, and is closely related to neuron morphology [128]. When SGK1 is overexpressed in the hippocampus, the total number of mature spines in dentate gyrus increases, and when SGK1 is reduced, the number of spines in CA1 increases, affecting synaptic structure and function in a region-specific manner depending on *SGK1* expression [129]. Similarly, increased *SGK1* expression in neurons promotes cell survival and inhibits apoptosis [130,131]. However, increased *SGK1* expression in glial cells leads to exacerbation of neuroinflammation and neuronal damage [132]. MECP2 directly and indirectly affects *SGK1* expression [133]. In particular, the absence of MECP2 prevents its binding to the proximal-promotor region of *SGK1*, thereby failing to suppress the expression of *SGK1* [134].

*IGFBP3* is directly regulated by MECP2, and *IGFBP3* overexpression owing to MECP2 deficiency is a hallmark of Rett syndrome in both mice and humans [135]. IGFBP3 is a binding protein for IGF-1 and IGF-2 and an important factor in cell growth and neuronal dendrite and axon elongation [136,137]. Thus, increased *IGFBP3* expression inhibits neurodevelopment. For example, *IGFBP3*-transgenic mice exhibit delayed brain growth and abnormal dendritic extension of neuronal cells [138]. As IGFBP3 binds IGF-1 and IGF-1 mimetics have shown therapeutic benefits in Rett syndrome, targeting the IGF system may be a potential strategy for Rett syndrome (see Section 5.4).

**Inhibitory Neurotransmission-Related Genes**

Key inhibitory neurotransmission-related genes, such as *DLX5* and *GABRB3*, are also transcriptionally regulated by MECP2. DLX5 regulates the expression of glutamate decarboxylase, a key enzyme for GABA synthesis, during the development of GABAergic neurons [139]. *DLX5* expression is regulated by MECP2, and thus *DLX5* is overexpressed in the absence of MECP2 [140]. However, *DLX5* expression also varies among Mecp2-deficient mice and is not consistently upregulated in the prefrontal cortex [141]. Further studies should determine whether this phenomenon is consistent across different brain regions and animal models. GABRB3 encodes the β3 subunit of the GABA receptor, which is a key receptor that mediates fast inhibitory neurotransmission in the brain, and is critical for maintaining excitation and inhibition (E/I balance), which is a mechanism underlying seizures [142,143]. MECP2 positively regulates *GABRB3* expression, and its deficiency contributes to E/I imbalance, a key symptom of Rett syndrome [142].

**Other Genes Regulated by MECP2**

FKBP5 regulates the function of glucocorticoid receptors and plays a key role in regulating stress hormones and synaptic plasticity. *FKBP5* expression is repressed by MECP2 [133,144], and is overexpressed in the Rett syndrome mouse model [133]. Thus, MECP2 deficiency causes abnormal regulation of stress and glucocorticoid signals during brain development.

All four known members of the inhibitor of DNA binding/differentiation subfamily (ID1, ID2, ID3, and ID4) are targets of MECP2 [145]. In *Mecp2*-dificent mice, the expression of ID protein was increased [145]. ID proteins regulate the fate of NSCs by influencing transcriptional programs important for differentiation. Specifically, they inhibit the activity of basic helix–loop–helix (bHLH) transcription factors, which play a key role in initiating neuronal differentiation by activating neuron-specific gene expression. However, the ID protein itself lacks a DNA-binding domain and thus acts as a dominant-negative regulator by forming a dimer with bHLH transcription factors to prevent DNA binding. Therefore, increased ID protein expression in the absence of MECP2 promotes NSC self-renewal and proliferation but suppresses neuronal differentiation [146,147,148]. Thus, MECP2 deficiency enhances NSC proliferation via increased expression of ID proteins, but reduces differentiation into neurons, contributing to the symptoms of Rett syndrome [102].

*FXYD1*, which encodes a membrane protein that regulates Na^+^-K^+^ ATPase activity important for neural function, is directly repressed by MECP2 [149]. In both patients with Rett syndrome and Mecp2-deficient mice, *FXYD1* is overexpressed in the prefrontal cortex, which reduced dendritic arborization and spine formation, decreased Na^+^-K^+^ ATPase activity, disrupted potassium homeostasis, and caused abnormal neuronal activity [149]. Interestingly, partial FXYD1 expression reduction by deleting one *Fxyd1* allele in Mecp2-deficient mice reverses the defects in neuronal branching and potassium regulation [150].

Early postnatal UBE3A expression is essential for proper maturation of neural circuits, including those in the striatum and hippocampus [151]. *UBE3A* mutations cause Angelman syndrome, where UBE3A deficiency disrupts synaptic development, neural circuit formation, and brain function during critical developmental stages [152]. UBE3A expression is also reduced in patients with Rett syndrome and Mecp2-deficient mouse models [153,154]. UBE3A expression is indirectly upregulated by MECP2, and its levels are reduced in Rett syndrome. Thus, UBE3A deficiency owing to MECP2 deficiency can help explain the pathophysiological overlap between Rett syndrome and Angelman syndrome [154].

Additionally, reduced MECP2 expression significantly impairs not only neuronal gene transcription but also mitochondrial function, including basal respiration, maximal respiration rate, and electron transport chain activity in neurons [32]. In addition, MECP2 appears to be involved in multiple metabolic processes, including lipid, nucleotide, glucose, and energy metabolism, as demonstrated in *Mecp2*-null mouse models [155].

Therefore, further studies are needed to elucidate the specific genes and proteins regulated by MECP2. Recent advances in artificial intelligence (AI)-based structure prediction tools, such as AlphaFold, allow us to assess whether specific MECP2 variants cause up- or down-regulation of target proteins and to predict potential binding interactions between MECP2 and specific proteins [156,157]. Leveraging these AI models will be crucial for elucidating the molecular interactome of MECP2. Future research should integrate AI-driven predictions with experimental validation to determine mutation-specific alterations in protein expression and to identify previously unrecognized molecular targets of MECP2.

#### 4.2.4. Neuronal Imbalance

In excitatory and inhibitory (E/I) neurons, MECP2 dysfunction causes structural deficits, including smaller soma size, shorter dendritic length, and fewer excitatory synapses. Such alterations ultimately impair E/I synaptic transmission [158].

MECP2 interacts with the Drosha complex, potentially affecting the processing of primary miR-199a (Pri-miR-199a) into precursor miR-199a (Pre-miR-199a). The pre-miR-199a produced in the nucleus undergoes processing in the cytoplasm to become mature miR-199a-5p/3p, which inhibits *SIRT1*, *HIF1a*, and *PDE4D*. These regulators negatively modulate mTOR signaling, which is linked to neurotransmission at excitatory synapses and neuronal cell growth. Therefore, reduced MECP2 decreased Drosha complex activity and reduced miR-199a levels, thereby enhancing mTOR inhibition and resulting in abnormal phenotypes of excitatory neurons, such as reduced soma size and decreased dendritic density [35].

Hdac1 and Hdac2 interact with Mecp2 and form a complex to bind to the promoter of Sap90/Psd95-associated protein 3 (*Sapap3*), a key component of excitatory synapse postsynaptic proteins [159,160]. Pre- and postnatal *MECP2* loss in the forebrain causes excessive grooming, a stereotypic behavior commonly observed in Rett syndrome [160]. *Mecp2* deletion or both *Hdac1* and *Hdac2* loss downregulates *Sapap3*, which leads to repetitive behavior. Reintroduction of *Sapap3* expression successfully rescues these behavioral abnormalities [158].

Taken together, MECP2 regulates excitatory synapse function by controlling miR-199a processing via the Drosha complex and repressing *Sapap3* expression through interaction with HDAC1/2. Its deficiency disrupts mTOR signaling and synaptic protein expression, leading to neuronal and behavioral abnormalities in Rett syndrome.

#### 4.2.5. Neuronal Types Affected by MECP2 Mutations

Cortical GABAergic neurons exhibit approximately 50% higher MECP2 expression level than non-GABAergic neurons, suggesting that MECP2 deficiency has a more significant effect on GABAergic neurons than on other neurons [161]. MECP2 loss leads to modifications in inhibitory intensity within the cortex, hippocampus, and brainstem [162,163]. MECP2 deficiency in GABAergic neurons leads to the loss of multiple functions such as social behavior, learning, memory, motor function, stereotyped behaviors, and sensorimotor gating. MECP2 deficiency also reduces presynaptic glutamic acid decarboxylase 1 (Gad1; GAD67) and glutamic acid decarboxylase 2 (Gad2; GAD65) levels, thus decreasing GABA production [161].

MECP2 deficiency reduced neurotransmitter levels in both dopaminergic and serotonergic neurons [164]. The role of the serotonergic system has been emphasized in relation to anxiety, repetitive behavior, and hyperactivity [165]. Particularly, in patients with Rett syndrome, low levels of serotonin (5-HT) have been reported, and deficiencies in serotonergic neurotransmission are associated with apnea, anxiety, and repetitive behaviors [164].

Dysfunctional dopaminergic activity is characterized by rigidity and movement abnormalities [23,166]. Reduced dopamine levels owing to MECP2 deficiency result in motor abnormalities in both mice and humans [164,167].

Both GABA and glycine are involved in neuronal development and regeneration [168,169]. Particularly, glycinergic neurons play a key role in the spinal cord and are co-localized with GABAergic neurons in cerebellar cortical interneurons. In the medulla oblongata, glycinergic neurons contribute to dual inhibitory synaptic transmissions in conjunction with GABA, playing a crucial role in regulating vital brainstem functions such as respiration. A recent study using Rett syndrome mouse models suggested that impaired GABAergic signaling appears to increase reliance on glycine-mediated inhibition [170].

#### 4.2.6. Cerebellar Cortex Dysfunction in Rett Syndrome

Cerebellar dysfunction and progressive cerebellar circuit loss are observed in Rett syndrome [110]. Rett syndrome is associated with Purkinje cell (PC) loss in the cerebellar cortex [171,172]. PCs are GABAergic and inhibitory, highly interconnected with up to 500 synaptic connections per cell, and contribute to motor coordination [173]. *MECP2* deletion in PCs increases PTP1B, a small-conductance calcium-activated potassium channel, and decreases signaling through TrkB, the receptor for BDNF, reducing intrinsic excitability. This ultimately contributes to autistic-like behaviors and impaired vestibulo-cerebellar motor learning [174]. Patients with Rett syndrome exhibit reduced cerebrum–cerebellum connectivity than the general population, with disrupted functional connectivity between the occipital lobe and cerebellum being implicated in the pathophysiology of autism spectrum disorder and schizophrenia. Additionally, decreased cerebellum–cerebrum/corpus callosum connectivity is associated with various behavioral and motor dysfunction phenotypes [110]. These reports indicate that MECP2 deficiency impairs cerebellar circuits and connectivity, contributing to the motor and behavioral symptoms of Rett syndrome.

#### 4.2.7. Hippocampal Dysfunction in Rett Syndrome

The hippocampus plays critical roles in learning and long-term memory formation [175]. In Mecp2-deficient mice, hippocampal neurons exhibit reduced dendritic complexity and spine density, accompanied by a decreased thickness of the CA1 region of hippocampus [176,177]. Furthermore, NMDA receptor maturation in the hippocampus is delayed, and synaptic plasticity is impaired [178]. Accordingly, patients with Rett syndrome exhibit reduced expression of metabotropic glutamate receptors, which are essential for learning and memory, than healthy individuals [179].

In Rett syndrome mouse models, theta-burst stimulation fails to induce LTP in CA1 hippocampal neurons. This impairment was accompanied by E/I imbalance and altered excitability in neurogliaform (NGF) interneurons, suggesting their involvement in Rett syndrome pathophysiology [180]. Additionally, *Mecp2* deficiency significantly decreases the frequency of spontaneous miniature excitatory postsynaptic currents in hippocampal neurons [181].

#### 4.2.8. Olfactory Bulb

Olfactory receptor neurons (ORNs) in mice are continuously replaced throughout life owing to postnatal neurogenesis [182]. NSCs located in the subventricular zone may contribute to this postnatal neurogenesis by migration through the rostral migratory stream to the olfactory bulb. MECP2 function loss results in an abnormal accumulation of immature ORNs and olfactory epithelium expansion [183,184]. Immature ORNs are characterized by neuron-specific tubulin (NST)-positive markers, while mature ORNs express olfactory marker protein (OMP). In typical conditions, the ratio of immature to mature ORNs remains relatively low (approximately 1.2–2), but this ratio is markedly elevated in Rett syndrome models (2.4–13.5). Additionally, OMP-positive ORN display abnormal dendritic morphology and dysmorphic cell bodies in patients with Rett syndrome [184].

#### 4.2.9. Autonomous Nervous System

Rett syndrome is characterized by dysfunction and dysregulation of the autonomic nervous system (ANS), which is considered a major cause of sudden death [185,186]. Rett syndrome features various characteristic respiratory patterns, including hyperventilation, apnea, apneusis, Valsalva maneuvers, breath-holding, and rapid shallow breathing, consistent with autonomic dysregulation [187,188,189]. Furthermore, in patients with Rett syndrome, even breathing that seems normal is actually more irregular than in healthy individuals, with increased average inspiratory flow rates, reduced expiratory times, and elevated respiratory frequency [190]. The activation of serotonin type 1A (5-HT1A) receptor, a key receptor involved in the termination of inspiration and located in the brainstem, promotes regular breathing and plays a role in homeostatic CO_2_ regulation [191].

In Rett syndrome, reduced serotonin (5-HT) levels decrease the activation of 5-HT1A receptors in the brainstem, resulting in dysregulation of ANS and contributing to the occurrence of apneas [192,193]. Heart rate regulation is also impaired in Rett syndrome, as indicated by a shortened R–R interval, corresponding to the ventricular depolarization phase of the cardiac cycle, and a weakened association between respiratory cycles and heart rate variability. In addition, unlike healthy individuals whose heart rate decreases during breath-holding, individuals with Rett syndrome show an increase in heart rate [190].

### 4.3. Glial Cells Affected by MECP2 Mutations and Rett Syndrome

Astrocytes are the most abundant type of glial cells in the brain and nervous system [194]. They support neuronal functions and respond to brain and neuronal damage, thereby helping the brain to maintain normal function [195]. Astrocytes are also connected to numerous synaptic sites and play a role in regulating the number and function of synapses [196]. Therefore, owing to their extensive roles in the nervous system, astrocyte dysfunction can significantly contribute to Rett syndrome pathophysiology, either directly or by disrupting neuronal synaptic function and homeostasis.

Although MECP2 expression is relatively low in astrocytes, it exerts substantial regulatory effects on their function and fate determination. NSCs differentiated from Rett syndrome iPSCs, which were generated from the fibroblasts of patients with Rett syndrome, show reduced neuronal differentiation potential and instead tend to differentiate more into astrocytes compared to wild-type NSCs [197]. Mecp2-null mice show a significant increase in GFAP and S100β, which are major markers of astrocytes [198]. This suggests that MECP2 plays a regulatory role in the expression of astrocyte-specific genes. MECP2 binds to the methylated promoter region of *GFAP* to regulate its expression, and MECP2 deficiency leads to GFAP overexpression [199]. Furthermore, *Mecp2* knockout or knockdown reduces astrocyte growth, and is proportional to the extent of Mecp2 expression reduction [200]. These findings indicate that Mecp2 deficiency or reduced expression can lead to astrocyte dysfunction, contributing to pathological outcomes.

Glutamate plays a crucial role in signal transmission by maintaining its proper levels [201]. When extracellular Glu concentration becomes excessively high, glutamate transporter EAAT1/GLAST and EAAT2/GLT-1 expression is suppressed [202]. Under normal conditions, EAAT1 and EAAT2 transport glutamate into astrocytes, where glutamine synthetase (GS) converts it into active neurotransmitters [202,203]. In *Mecp2*-deficient mice, elevated extracellular glutamate levels suppress EAAT1/EAAT2 expression and increase GS expression, resulting in dysregulation of extracellular glutamate concentration [204]. This demonstrates that Mecp2 deficiency leads to abnormal regulation of the glutamate metabolic pathway in astrocytes, contributing to Rett syndrome pathology.

Interestingly, long-term co-culture of *Mecp2*-null astrocytes with wild-type astrocytes reduced Mecp2 expression in the wild-type astrocytes. This non-cell-autonomous effect is mediated by Connexin-43 (Cx-43)-based gap junctions [200]. When these gap junctions are blocked by Cx-43 siRNA, this effect is diminished, suggesting that targeting gap junctions might be a potential therapeutic strategy for Rett syndrome phenotypes.

In neurons and glial cells of Rett syndrome cerebral organoids, mitochondrial length and area were reduced compared to those in the control group, and the alteration was particularly significant in glial cells [205,206,207]. Additionally, Rett syndrome astrocytes show an overall decrease in oxygen consumption rate and overall ATP production [206]. Accordingly, Rett syndrome astrocytes exhibit abnormal mitochondrial and metabolic function, along with increased amino acid metabolism to compensate for the energy deficit. Interestingly, abnormal mitochondria in Rett syndrome astrocytes can be transferred to neurons [208], which cause neuronal hyperexcitability and neuronal dysfunction [206,209].

Oligodendrocytes also express MECP2 [210] and oligodendrocyte-specific proteins, such as crystallin B and S100a13, were abundant in the post-mortem brains of patients with Rett syndrome [211]. Conditionally knocked-out mice with *Mecp2* deficiency specifically in oligodendrocyte lineages demonstrate severe hindlimb clasping phenotypes, which are typically observed in Rett syndrome models [212]. MECP2 is also essential for the oligodendrocyte maturation from oligodendrocyte progenitor cells. When differentiation is induced in MECP2-mutated NSCs, oligodendrocyte differentiation rate is decreased [213]. However, glial differentiation mechanisms may vary dependent on species, brain region, and environmental factors [214]. In addition, MECP2 is associated with oligodendrocyte survival and myelination [215].

Collectively, MECP2 in glial cells, including astrocytes and oligodendrocytes, contributes to the neurological manifestations of Rett syndrome and glial cells play an active role in disease pathology beyond neuronal dysfunction directly or indirectly.

### 4.4. Other Organs Affected by MECP2 Mutation and Rett Syndrome

Genetic disorders usually affect multiple organ systems [216] MECP2 is abundantly expressed in not only the brain but also the heart, kidneys, lungs, and spleen, whereas its expression is minimal in the liver, stomach, and small intestine [99]. As an epigenetic regulator, MECP2 plays a critical role in cardiovascular function. Prolonged QTc intervals have been observed in some patients with Rett syndrome and Mecp2-null male mice [217]. MECP2 is also involved in cardiac development. *Mecp2*-deficient mouse ESCs show severely impaired development into cardiovascular progenitors, and *Mecp2*-null mice display dysregulated cardiac gene expression and structural abnormalities in the myocardium [218].

Urinary dysfunction is reported in some patients with Rett syndrome. *Mecp2*-deficient mice display frequent urination, reduced void volume, and signs of renal failure owing to urethral obstruction [219]. Interestingly, MECP2 is upregulated during ischemia–reperfusion-induced acute kidney injury, where it exerts a protective effect by suppressing the IL-6/STAT3 axis. On the contrary, MECP2 deficiency worsens renal injury and promotes cell death, inflammation, and fibrosis [220]. These findings indicate that MECP2 functions not only as a neuronal gene but also as a key kidney function regulator.

MECP2 deficiency also impacts the immune system within the spleen. MECP2 is expressed in spleen-resident macrophages in mice, and peripheral macrophage populations are reduced in *Mecp2*-null mice than in controls, suggesting that Mecp2 deficiency may affect the function of the peripheral immune system, such as the spleen [221]. Meanwhile, splenocytes from *Mecp2*-deficient mice with experimental autoimmune encephalomyelitis maintained a proinflammatory profile from acute to chronic stages upon in vitro autoantigen restimulation, indicating that Mecp2 loss may lead to excess inflammatory responses [222].

Respiratory dysfunction caused by MECP2 deficiency is primarily attributed to impaired neural regulation within the brainstem. In *Mecp2*-deficient mice, neurotransmitter expression related to swallowing and airway protection is reduced in the brainstem [223]. However, respiratory dysfunction in Rett syndrome is also associated with morphological abnormalities of the lungs. High-resolution CT scans of the lungs in females with MECP2 mutations revealed various abnormalities, including centrilobular nodules, bronchial wall thickening, patchy ground-glass opacities, and bronchiectasis in about half cases [224]. Additionally, pulmonary dysfunction also contributes to respiratory abnormalities. Conditional *MECP2* knockout in alveolar epithelial type II cells caused abnormal lung lipid profiles and respiratory symptoms. These respiratory abnormalities were distinct from those observed when *Mecp2* was deleted only in brainstem neurons [225].

## 5. Rett Syndrome Treatment

### 5.1. AAV9-Based Gene Replacement Therapy

Wild-type *MECP2* gene delivery has been extensively studied as a treatment for Rett syndrome [226]. Injecting a self-complementary AAV9 vector expressing a codon-optimized Mecp2 (scAAV9-MCO) improved symptoms and survival in *Mecp2*-deficient mice but caused toxicity at high doses [227,228]. To address these issues, second-generation vectors with extended *Mecp2* promoters and regulatory 3′-UTR elements reduced liver toxicity, enhanced brain delivery, and improved outcomes in neonatal *Mecp2*-deficient mice [229]. AAV-PHP.B demonstrated excellent blood–brain barrier penetration with high specificity for both neurons and glial cells in adult mice [230,231]. Intravenous injection of an AAV-PHP.eB vector expressing instability-inducing MECP2 (iMECP2) improved motor function and lifespan without liver toxicity [232]. The capsid-modified AAV.CAP-B10 achieved strong transgene expression in multiple brain regions of mice and marmosets [233]. Additionally, miniMECP2 gene therapy (TSHA-102) with a miRNA-responsive element improved respiration, weight, survival, and motor skills in Rett syndrome mice [234].

Although *MECP2* gene delivery techniques for the treatment of Rett syndrome have advanced, there is still a lack of research on how long the transgenic MECP2 is expressed and when its expression may cease. To achieve clinical improvement, further studies are needed to define the optimal timing for intervention.

### 5.2. MECP2 Gene Editing and Xi Reactivation

The adenosine deaminases acting on RNA 2 (ADAR2) catalyzes adenosine-to-inosine conversion in double-stranded RNA, which is one of the most common forms found in brain mRNA [235,236]. Sinnamon et al. employed an AAV vector with hyperactive ADAR2 for RNA editing, restoring MECP2 expression in cultured hippocampal neurons and in the hippocampus and brainstem of Rett syndrome mouse models [237,238,239]. Meanwhile, in females with Rett syndrome, random XCI results in approximately 50% cells expressing mutant MECP2 and the remainder expressing normal MECP2 [240]. Therefore, X chromosome reactivation has been considered a potential therapeutic approach for Rett syndrome. Combined treatment with an antisense oligonucleotide targeting Xist and a DNA methylation inhibitor significantly increased *Mecp2* expression from the Xi in mouse fibroblasts [241]. Subsequent studies pharmacologically inhibited X chromosome inactivation factors (XCIFs) and reactivated the Xi-linked *Mecp2* in mouse cortical neurons in vivo [242]. However, its effects on Rett syndrome phenotypes remain to be determined. Recently, multiplex epigenome editing restored MECP2 expression that follows restoration of neuronal soma size and electrophysiological function in human Rett syndrome ESCs and their derivative neurons [243]. However, further validation in living animal models is needed to evaluate their clinical relevance.

### 5.3. Anti-Inflammation and Immunomodulation

MECP2 regulates microglial responses to inflammatory stimuli [221]. In *MECP2*-null microglia, increased glutamate secretion causes neurotoxicity [121]. Both *Mecp2*-deficient mice and patients with Rett syndrome show excess inflammation and oxidative stress [244,245]. Dendrimer-N-acetyl cysteine (D-NAC) selectively targets microglia and improves neurological and behavioral functions [246]. Inhibiting the overactive inflammatory RIPK1 kinase pathway in *MECP2*-deficient microglia reduces oxidative stress and inflammatory cytokine release, improving neurological function [247]. In *Mecp2*-null mice, the downstream gene *Irak1* is overexpressed, which significantly activates the NF-κB pathway. Therefore, lowering NF-κB expression could enhance the dendritic complexity of cortical callosal projection neurons (CPNs) and substantially prolong their lifespan [122].

### 5.4. Pharmacological Therapy

Trofinetide (Daybue™), an oral solution at a concentration of 200 mg/mL and a synthetic analog of the N-terminal tripeptide of insulin-like growth factor 1 (IGF-1; Gly–Pro–Glu (GPE)), was the first drug approved by the United States Food and Drug Administration (FDA) for the treatment of Rett syndrome in March 2023 [248]. A phase 3 trial in 187 female patients aged 5–20 showed significant improvements in behavior, clinical assessment, communication, and symbolic behavior [248]. Trofinetide improves the structure and function of synapses, reduces neuroinflammation and oxidative stress, modulates apoptosis, and promotes neuronal homeostasis, with a longer half-life than GPE [249]. Trofinetide showed clinically meaningful improvements in Rett Syndrome Behavior Questionnaire (RSBQ) and Clinical Global Impression–Improvement (CGI-I) scores in patients with Rett syndrome. Adverse effects were mainly limited to mild vomiting and diarrhea. Overall, its favorable safety profile and demonstrated efficacy position it as an important therapeutic option for Rett syndrome [250].

In *MECP2*-mutant neurons, protein production is decreased, a phenomenon linked to dysregulated AKT/mTOR signaling [32]. The AKT/mTOR pathway can be activated by inhibiting PTEN, a negative regulator of PI3K, increasing neuronal soma size and enhancing neurite arborization [251,252]. Through BDNF and IGF-1 treatment, Li et al. observed an increase in protein synthesis and the recovery of AKT/mTOR signaling [32]. Activation of AKT/mTOR signaling after growth factor administration ameliorates disease phenotypes, such as reduced soma size, reduced neurite complexity, and reduced action potential rates, in *MECP2*-mutant human neurons.

MECP2 deficiency contributes to Rett syndrome pathology through changes in NMDA receptor (NMDAR) expression [253]. Administering the NMDAR antagonist ketamine alleviated motor and respiratory deficits and extended survival in Rett syndrome models [254]. Although MECP2 acts as a transcriptional repressor of BDNF [255], BDNF expression progressively declined in the brainstem and nodose ganglion of Mecp2-deficient mice [256]. Given the importance of these regions in cardiopulmonary homeostasis [257], reduced BDNF may underlie severe respiratory dysfunction. The ampakine drug CX546, which enhances glutamatergic AMPA receptor activation, increased BDNF levels, leading to restored normal respiratory rate and ventilation in mice [256].

Increased TrkB activity may help restore abnormal movements of PCs caused by MECP2 deficiency, as well as synapse formation and structural defects in neurons [258,259,260]. A 5-HT1A agonist (NLX-101) can reduce irregular breathing in Rett syndrome by acting on the brainstem [193,261,262]. Improved respiration is mediated through GIRK channel activation, independently of neurotransmitter release mechanisms [193,261,262].

### 5.5. Other Therapeutic Approaches

Cell-based therapies, neurostimulation, and environmental interventions have also been studied as potential treatments for Rett syndrome. Co-culturing Mecp2-deficient neurons with NPCs restored neuronal morphology and synaptic function [263]. In addition, NPC transplantation to the cerebellum improved memory and motor functions in mouse models [263]. Transcriptomic analysis revealed that activation of interferon γ (IFNγ) may be a key molecular mechanism underlying the improved brain function following NPC transplantation.

Forniceal deep brain stimulation (DBS), an invasive intervention, improved hippocampal LTP and neurogenesis in Rett syndrome mouse model, thereby restoring contextual fear memory and spatial learning [264]. Beyond the hippocampus, forniceal DBS also activates neural networks within limbic structures involved in emotion and memory [265].

## 6. Conclusions

Rett syndrome is a complex neurodevelopmental disorder caused by mutations in the *MECP2* gene, leading to widespread epigenetic dysregulation and impaired neuronal and glial function. MECP2 is a global transcription factor that affects chromatin structure, alternative splicing, and miRNA processing, which in turn affect neurodevelopment, synaptic homeostasis, and cellular metabolism. These abnormalities affect various parts of the brain, including the cortex, hippocampus, cerebellum, and brainstem, as well as extracerebral areas, such as the heart, kidneys, spleen, and lungs.

We present strategies to understand Rett syndrome from various angles, including the molecular mechanisms of MECP2, MECP2-related mosaicism, and post-translational modifications. These insights have fueled the development of targeted therapeutic strategies, including AAV-based gene replacement, RNA editing, X chromosome reactivation, anti-inflammatory approaches, and pharmacological interventions such as Trofinetide, ketamine, and growth factors. Although various treatment strategies have been proposed, the MECP2 mosaicism and developing approaches that specifically target cells with abnormal MECP2, while sparing normal cells, should be considered to ensure safety and long-term efficacy. Future research should also focus on improving human-relevant disease models, developing personalized genetic and drug therapies, and exploring ways to overcome mosaicism. Additionally, MECP2 deficiency affects not only various cell types in the brain but also the function of multiple peripheral organs. Therefore, further research is needed to characterize tissue- and cell-type-specific responses to MECP2-based therapies.

## Abbreviations

The following abbreviations are used in this manuscript:

ADAR2	Adenosine deaminases acting on RNA 2
ANS	Autonomous nervous system
BDNF	Brain-derived neurotrophic factor
CREB1	cAMP-responsive element-binding protein 1
CRF	Corticotropic releasing factor
CTD	C-terminal domain
DAN	Dorsal attention network
DBS	Deep brain stimulation
ERK	Extracellular signal-regulated kinase
GABA	γ-aminobutyric acid
GABA_A_	γ-aminobutyric acid a
GFAP	Glial fibrillary acidic protein
GIRK	G protein-gated inwardly rectifying potassium channel
GO	Gene Ontology
GPE	Glycine-Proline-Glutamate
GS	Glutamine synthetase
HAT	Histone acetyltransferase
HDAC	Histone deacetylases
ID	Intervening domain
IGF	Insulin-like growth factor
LTP	Long-term potentiation
MBD	Methyl-CpG-binding domain
MECP2	Methyl-CpG-binding protein 2
NCoR	Nuclear receptor co-repressor
NGF	Neurogliaform
NID	NCoR–SMRT interaction domain
NMDA	N-methyl-D-aspartate
NMDAR	NMDA receptor
NPC	Neural Progenitor Cell
NSC	Neural stem cells
OMP	Olfactory marker protein
ORN	Olfactory receptor neurons
PC	Purkinje cell
PTEN	Phosphatase and tensin homolog
PTZ	Neural progenitor cells
SIN3A	Switch-independent 3A
SMRT	Silencing mediator of retinoic acid and thyroid hormone receptor
TrkB	Tropomyosin receptor kinase B
TRD	Transcriptional repression domain
TSS	Transcription start site
WT	Wild type
XCI	X chromosome inactivation
